# N-Substituted Phenylhydrazones Kill the Ring Stage of *Plasmodium falciparum*

**DOI:** 10.1155/2024/6697728

**Published:** 2024-02-13

**Authors:** Cedric Dzidzor Kodjo Amengor, Prince Danan Biniyam, Abena Amponsaa Brobbey, Francis Klenam Kekessie, Felix Kwame Zoiku, Sherif Hamidu, Patrick Gyan, Billy Mawunyo Abudey

**Affiliations:** ^1^Department of Pharmaceutical Chemistry, School of Pharmacy, University of Health and Allied Sciences, Ho, Ghana; ^2^Department of Pharmaceutical Chemistry, Faculty of Pharmacy and Pharmaceutical Sciences, Kwame Nkrumah University of Science and Technology, Kumasi, Ghana; ^3^Department of Chemistry and Biochemistry, The University of Southern Mississippi, Hattiesburg Campus, 118 College Drive, Hattiesburg, USA; ^4^Department of Epidemiology, Noguchi Memorial Institute for Medical Research, College of Health Sciences, University of Ghana, P.O. Box LG 581, Legon, Accra, Ghana; ^5^Department of Clinical Pathology, Noguchi Memorial Institute for Medical Research, College of Health Sciences, University of Ghana, P.O. Box LG 581, Legon, Accra, Ghana

## Abstract

Antimalarial resistance has hampered the effective treatment of malaria, a parasitic disease caused by *Plasmodium* species. As part of our campaign on phenotypic screening of phenylhydrazones, a library of six phenylhydrazones was reconstructed and evaluated for their *in vitro* antimalarial and in silico receptor binding and pharmacokinetic properties. The structures of the phenylhydrazone hybrids were largely confirmed using nuclear magnetic resonance techniques. We identified two compounds which exhibited significant antimalarial potential against the ring stage (trophozoite) of 3D7 chloroquine-sensitive (CS) strain and DD2 chloroquine-resistant (CR) strains of *Plasmodium falciparum* with monosubstituted analogs bearing *meta* or *para* electron-donating groups showing significant activity in the single-digit micromolar range. Structure activity relationship is presented showing that electron-donating groups on the substituent hydrophobic pharmacophore are required for antimalarial activity. Compounds PHN6 and PHN3 were found to be the most potent with pIC_50_s (calculated form *in vitro* IC_50_s) of 5.37 and 5.18 against 3D7 CS and DD2 CR strains, respectively. Our selected ligands (PHN3 and PHN6) performed better when compared to chloroquine regarding binding affinity and molecular stability with the regulatory proteins of *Plasmodium falciparum*, hence predicted to be largely responsible for their *in vitro* activity. Pharmacokinetic prediction demonstrated that the phenylhydrazones may not cross the blood-brain barrier and are not *P*-glycoprotein (P-gp) substrates, a good absorption of 62% to 69%, and classified as a category IV compound based on toxicity grading.

## 1. Introduction

**Figure 1:**
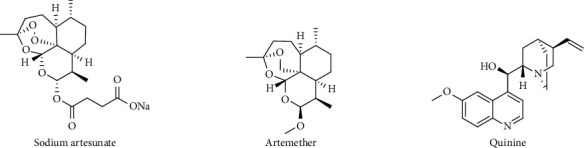
Some current antimalarials with *Plasmodium falciparum* resistance concerns.

While the world is still recovering from the COVID-19 epidemic, *falciparum* malaria, which is still the most deadly parasitic illness transmitted by the phlebotomine female anopheles mosquito, is emerging as one of the worst global health issues in history [1]. There have been ongoing efforts to hasten the development of antimalarial drugs due to the growth of *Plasmodium falciparum* strains that are resistant to artemisinin and other existing chemotherapeutic agents [2]. Due to the rising rates of morbidity and mortality brought on by malaria, *Plasmodium* has evolved resistance to the majority of therapeutic drugs authorised for the treatment of the disease. These include artemisinin-based combination therapies (ACTs) (Figure 1) as first-line regimen for early-stage *P. falciparum* malaria.

This has made it necessary as a global concern to search for novel antimalarial drugs [3, 4].

Aspartic protease enzymes (plasmepsins IV and II) and cysteine protease falcipain-2 of *Plasmodium falciparum*, which play crucial roles in the parasite's survival as major peptide hydrolases within the haemoglobin degradation pathway and an essential role in this critical nutrient acquisition pathway, respectively, are among the promising new targets in antimalarial drug design. As a result, these proteins are among the most desirable targets for the development of antimalarial drugs [5–7]. Until now, falcipain-2 and plasmepsin (II and IV) inhibitors may prevent the parasite from making proteins, which would prevent it from absorbing nutrient [6, 7]. Therefore, it is advantageous for novel antimalarial drugs to be able to target these associated proteins and have a good pharmacokinetic profile. A class of imine medicines known as phenylhydrazones has been shown to have powerful antimalarial properties by blocking the heme to haemazoin pathway and killing *P. falciparum* [8, 9]. These include novel target proteins for antimalarials including the *Plasmodium* kinome, food vacuole, cysteine proteases, and aminopeptidases. Older targets include plasmepsins which are still relevant in antimalarial drug discovery because they play a key role in the breakdown of haemoglobin for the parasite's survival [9]. By blocking important *Plasmodium falciparum* enzymes, hydrazones have antimalarial action [9, 10].

**Figure 2:**
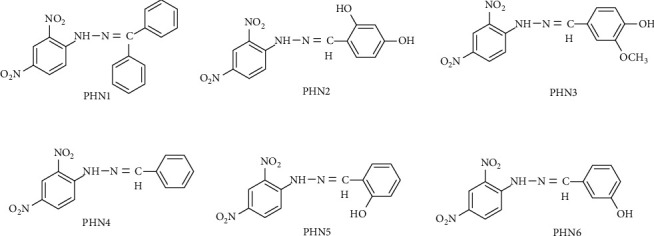
Structures of the resynthesized N-substituted prospective antimalarial compound.

Herein, we have docked synthetic ligands (phenylhydrazones) with antimalarial properties on falcipain-2 protease, plasmepsin II, and plasmepsin IV receptors to gain insight into the drug-receptor interactions in order to further understand the biology of parasite. The resynthesis, biological activity, and *in silico* profile of the N-substituted phenylhydrazones (Figure 2) are all reported in this current work.

This is a component of a future effort to optimize hit-to-lead conversion by exploring the chemical space around the core of these compounds.

## 2. Materials and Methods

### 2.1. Chemistry

Using a Bruker TopSpin 3.2 NMR 400 MHz spectrometer (NM 103508-10, Germany) and TMS as the internal standard (chemical shifts in) and deuterated chloroform as the lock solvent, ^1^H, ^13^C nuclear magnetic resonance (NMR), and DEPT-Q analysis were carried out on the compounds. In Hz, coupling constants (*J*) are expressed. The general procedure was according to [11], and the synthesized compounds were purified by recrystallization from absolute ethanol (96% *v*/*v*). All compounds' scanned spectra are displayed in Figure S1 to Figure S12.

#### 2.1.1. Synthetic Data

**Figure 3:**
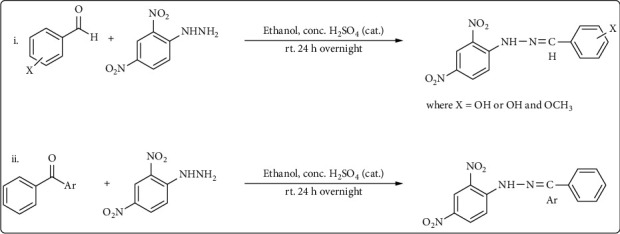
General scheme (i for aldehyde, ii for ketone) for the synthesis of the phenylhydrazones.

All the compounds have been reconstructed, and their spectroscopic data was found to be consistent with literature [11]. General scheme is in Figure 3.

*(1) PHN1: (2,4-Dinitrophenyl)-2-(diphenylmethylene) Hydrazine*. (0.84 g, 85 % w/w) presented as a brick red solid. Mpt: 140-143 oC; UV-Vis (MeOH) *λ*_max_: 382 nm; Infra-red (neat) *υ*_max_: 3382, 3286, 1586, 848, 614 cm^−1^. *δ*_H_ (400 MHz; CDCl_3_): 11.24 (1H (C1), s, NH), 9.09-9.10 (1H (C3), s, ArH), 8.41 (1H, (C5) d, *J* =2.4, ArH), 8.37-8.38 (1H (C6), m, ArH), 7.66-7.72 (5H, (C1', C2', C3', C2”, C6”) m, ArH), 7.35 (3H, C(3”), C(4”), C(5”), m, ArH), 7.57 (2H (C4', C6'), m, ArH). *δ*_c_ 131.9,130.5, 130.4,130.0,129.9,128.5,128.1, 127.9 (Ar).

*(2) PHN2: 4-(2-(2,4-Dinitrophenylhydrazono) Methyl) Benzene-1,3-diol*. (0.83 g, 72 % w/w) was produced as a dark red amorphous solid. Mpt: 270-273 oC; UV-Vis (MeOH) *λ*_max_: 218 nm and 400 nm; Infrared (neat) *υ*_max_: 3350 (broad, OH), 3090 (sharp, C=C**H**), 1620 (sharp, C=C), 1570 (N=N) cm^−1^. *δ*_H_ (400 MHz; CDCl_3_): 8.87 (1H (C1'), s, ArOH), 8.81 (1H (C1), s, NH), 8.35-8.36 (1H (C3'), s, ArOH), 8.33-8.35 (1H (C3), s, ArH), 7.95-7.97 (1H, s, N=C**H**), 7.64 (1H (C5), d, *J*=12.0, ArH), 7.66 (1H (C6, d, *J*=12.0, ArH), d, *J*=12.0, ArH), 6.36-6.38 (2H (C2', C5', m, ArH), d, *J*=8.0, ArH), 6.32 (1H (C4'), d, *J*=8.0, ArH). *δ*_c_ 161.8, 159.1, 148.2, 144.6, 136.7, 130.1, 129.3, 128.8, 123.6, 116.9, 112.0, 108.0, 102.0. These parameters were also consistent with literature [11]

*(3) PHN3: 4-(2-(2,4-Dinitrophenyl) Hydrazone) Methyl)-2 Methoxyphenol*. (0.83 g, 70 %w/w) presented as a bright red amorphous solid. R_f_ (Pet. ether 70 %: EtOAc 30 %): 0.65. Mpt: 268-270 oC. UV-Vis (MeOH) *λ*max: 218 nm and 392 nm. Infrared (neat) *υ*max cm ^−1^: 3330 (broad, OH), 3210 (sharp, NH), 3080 (sharp, C=CH), 1620 (sharp, C=C), 1570 (N=N); ^1^H NMR (400 MHz, CDCl_3_) *δ*H 11.56 (1H, s, NH), 9.66 (1H, H-C4', s, ArOH), 8.85-8.87 (1H, H-C3, s, ArH), 8.37 (1H, H-C5, s, ArH), 8.35 (1H, H-C7, s, N=CH), 8.34 (1H, H-C6, d, *J* = 4.0, ArH), 7.20-7.40 (1H, H-C2', d, *J* = 4.0, ArH), 7.18-7.19 (1H, H-C6', d, *J* = 8.0, ArH), 6.87-6.89 (1H, H-C5', d, *J* = 12.0, ArH), 3.87 (3H, H-C4', Ar-OCH_3_). ^13^C NMR (400 MHz, CDCl_3_) *δ*c 150.6, 150.1, 148.6, 144.9, 137.1, 130.1, 125.2, 123.1, 123.0, 117.0, 116.1, 110.2 (Ar-C), 56.2 (**C**H_3_) ppm.

*(4) PHN 4: (Z)-2-(2,4-Dinitrophenyl)hydrazono) Methylphenol*. (1.09 g, 92 %w/w) as an orange solid. Mpt: 176-180 oC; UV-Vis (MeOH) *λ*_max_: 386 nm; Infra-red (neat) *υ*_max_: 3334, 3267, 3059, 1583, 759 cm^−1^. *δ*_H_ (400 MHz; CDCl_3_): 11.25 (1H (C3'), s, ArOH), 9.98 (1H (C1), s, NH), 9.11 (1H C(3), s, ArH), 8.34-8.36 (1H (C7), s, N=C**H**), 8.33-8.36 (1H (C5), d, *J*=4.0, ArH), 8.23 (1H (C6), d, *J*=4.0, ArH), 7.58-7.61 (1H (C5'), m, ArH), 7.31-7.35 (1H (C1'), m, ArH), 7.24-7.26 (1H (C6'), m, ArH), 6.91-7.01 (1H (C2'), m, ArH). *δ*_c_ 157.9, 151.3, 132.9, 131.4, 130.6, 123.7, 120.3, 117.2, 116.9, 115.3 (Ar)

*(5) PHN5: 1-Benzylidene-2-(2,4-dinitrophenyl)hydrazine*. (0.97 g, 75 % w/w) as a bright orange solid. Mpt: 178-181 oC; UV-Vis (MeOH) *λ*_max_: 224 nm and 378 nm; Infra-red (neat) *υ*_max_: 3337, 3283, 3100, 1618, 1584 cm^−1^. *δ*_H_ (400 MHz; CDCl_3_): 11.24 (1H (C1), s, NH), 9.09 (1H (C3), s, ArH), 8.30-8.31 (1H (C7), s, N=CH), 8.28 (1H (C6), m, ArH), 8.02-8.06 (2H (C1', C5') m, ArH), 7.39-7.40 (3H (C2', C3', C4'), m, ArH); *δ*_c_ 147.9, 131.0, 130.0, 129.0, 127.6, 123.5, 116.8 (Ar).

*(6) PHN6: 3-(2-2(4-Dinitrophenyl) Hydrazono) Methylphenol*. (0.76 g, 64 % w/w) as a bright red solid. Mpt: 277-280 oC; UV-Vis (MeOH) *λ*_max_: 392 nm, Infra-red (neat): 3420, 3257, 3116, 1607, 1584 cm^−1^. *δ*_H_ (400 MHz; CDCl_3_): 11.56 (1H (C1), s, NH), 10.04 (1H (C2'), s, ArOH), 8.88 (1H (C3), s, ArH), 8.86 (1H (C7), s, N=CH), 8.35-8.37 (1H (C5), d, *J*=8.0, ArH), 8.08-8.34 (1H (C6), d, *J*=12.0, ArH), 8.05 (1H (C5'), m, ArH), 7.66 (1H (C1') s, ArH), 7.14 (1H (C4'), m, ArH), 6.87-6.89 (1H (C3'), m, ArH). *δ*_c_ 160.4, 150.5, 145.8, 144.9, 137.1, 130.2, 130.1, 129.8, 129.5, 123.5, 125.2, 117.1, 116.4 ppm.

### 2.2. Computational Methods

#### 2.2.1. Molecular Docking and Molecular Dynamics

The crystal structures of falcipain-2 protease (PDB ID: 6SSZ), plasmepsin II (PDB ID: 1LF3), and plasmepsin IV (PDB ID: 1LS5) were retrieved from Research Collaboratory for Structural Bioinformatics PDB (https://www.rcsb.org/pdb) using UCSF Chimera. These are essential proteins for the survival of the parasite as mentioned in the introduction for this study. The Dock Prep option available in UCSF Chimera was employed to prepare each of the proteins using the default parameters. This option makes it possible to delete solvent molecules, repair truncated side chains, add hydrogen, and assign charges to protein structures. The prepared proteins were saved in pdb format for easy recognition by the AutoDock tools and Swiss-PDB-Viewer. The ligands were drawn using ChemDraw Ultra 2D and then transformed into their 3D forms using ChemDraw Ultra 3D software. Polar hydrogens and Gasteiger charges were added to each ligand in UCSF Chimera. Avogadro-1.2.0 and Swiss-PDB-Viewer 4.1.0 were used in the energy minimization of the ligands and the proteins, respectively. The determinants of ligand binding were explored by molecular docking in AutoDock Vina extended into UCSF Chimera [12]. Plm II (PDB ID: 1LF3) was used in method validation of molecular docking by redocking native ligand (EH58) to the binding pocket of the Plm II. The grid coordinates and box dimensions used for the local docking were obtained by creating a receptor grid around the binding pocket of each of the prepared proteins, whereas in the blind docking, the whole protein was enclosed into the grid box.

Each docking experiment was repeated in four technical runs to ensure reproducibility, and in each run, nine poses were generated and ranked according to their binding energies by AutoDock Vina [13]. Selection of the best poses was based on ligands' binding energy and the ability of the ligands to form optimal interactions with active site residues of the target proteins [13]. The root mean square deviation of the redocked ligand was calculated in PyMOL. Protein-ligand interactions of the redocked outputs were visualized in PyMOL and PoseView program. The ADMET properties of the ligands were computed in SwissADME and Pro Tox II using their canonical smiles.

Molecular dynamic simulation study was also carried out to ensure the stability of the best protein-ligand complex. We performed the simulation using GROMACS version 2023.2 via Ubuntu 2022.04.2 LTS on a multiple CPU core computer. The topology file of the protein was generated using gmx pdb2gmx tool with the CHARMM36 all-atom force field and CHARMM-modified TIP3P water model [14]. Prior to this step, Charmm36-july2022 force field for GROMACS was downloaded via Mackerel lab online server. Since GROMACS does not have an internal module for ligand topology file preparation, we relied on CHARMM general force field online server as an external tool to generate the ligand topology file [13, 14]. A complex file was then generated by merging the topology file of the ligand and the protein. The generated complex was further solvated with TIP3P water model and neutralized with sodium and chloride ions in a dodecahedral box. CHARMM36 force field was employed to minimize the energy of all the atoms in the dodecahedral box using steepest decent algorithm for 50,000 steps. This was followed by heating the system, NPT (constant number of particles, pressure, and temperature) equilibration at 1 atm for 100 ps, and NVT (constant number of particles, volume, and temperature) equilibration of the system at 300 K for 100 ps. Linear Constraint Solver (LINCS) algorithm was used to constrain the covalent bonds, and long-range electrostatic interactions were calculated using particle mesh Ewald (PME) method. Three-dimensional periodic boundary conditions were set in all case, and an MD production run of 30 ns was performed. The coulomb and van der Waal's cutoffs were set to 1.2 nm, and the time step was defined as 2 fs. The coordinate trajectories were written at 10 ps intervals [14].

#### 2.2.2. ADME Prediction and Toxicity

Physical and chemical descriptors of the compounds were computed using SwissADME web tool suite. Toxicity analysis of the compounds was carried out in Pro Tox II, a free online virtual lab for prediction of toxicity of compounds [15].

### 2.3. Biology

The SYBR green and cytotoxicity assay procedure to determine the potency of the phenylhydrazones was according to [16].

#### 2.3.1. Preparation of Compounds

One-milligram (1 mg) powder of each compounds was weighed and transferred into dimethylsulphoxide (DMSO) (1 ml of 0.5% *v*/*v*) to yield a stock concentration of 1000 *μ*g/ml. The stock solution was vortexed well to dissolve the compounds and later filtered through 0.2 *μ*m pore filter unit and then stored in a -20°C freezer until use. The stock products were diluted 10-fold to obtain a working solution of 100 *μ*g/ml. This working solution was further serially diluted 9-fold to obtain the concentrations 100 *μ*g/ml, 50 *μ*g/ml, 25 *μ*g/ml, 12.5 *μ*g/ml, 6.25 *μ*g/ml, 3.13 *μ*g/ml, 1.56 *μ*g/ml, 0.78 *μ*g/ml, and 0.39 *μ*g/ml.

#### 2.3.2. Parasite Culturing and Preparation

The efficacy of compounds on asexual parasite stages was tested on the 3D7 chloroquine-sensitive strain of *Plasmodium falciparum* and DD2 chloroquine-resistance strain which were obtained from the Department of Immunology of Noguchi Memorial Institute for Medical Research, University of Ghana. Continuous *P. falciparum* asexual cultures were maintained *in vitro* in an atmosphere of 93% N_2_, 4% CO_2_, and 3% O_2_ at 37°C in complete medium (CM) (10.44 g/liter Roswell Park Memorial Institute Medium (RPMI) 1640, 5.94 g/liter N-2-hydroxyethylpiperazine-N-2-ethane sulfonic acid (HEPES), 5 g/liter AlbuMAX II, 50 mg/liter hypoxanthine, and 2.1 g/liter sodium bicarbonate). Parasites were cultured in O+ RBCs and maintained in the incubator with daily media change until a parasitemia of more than 5% ring stages was obtained. The culture was then treated with 5% sorbitol to obtain synchronized ring stage, and parasite growth was monitored for some few days by estimating percentage (%) parasitemia using Giemsa-stained slides and light microscope with 100x magnification until parasitemia of more than 5% was recorded. Parasite suspension of 2% hematocrit with 1% parasitemia was prepared using uninfected blood to make a total of 14 ml in a complete culture medium for the plating [16]. Compound plating and SYBR green assay were according to [16].

#### 2.3.3. Compound Plating and Assay

One hundred (100) microliters of each nine dilutions (100 *μ*g/ml, 50 *μ*g/ml, 25 *μ*g/ml, 12.5 *μ*g/ml, 6.25 *μ*g/ml, 3.13 *μ*g/ml, 1.56 *μ*g/ml, 0.78 *μ*g/ml, and 0.39 *μ*g/ml) was plated in duplicate in a 96-well coastal plate. 15 ng/ml of artesunate was serially diluted and plated alongside with the compounds as a standard antimalarial control drug. One hundred of parasite mix with 2% hematocrit and 1% parasitemia were added to each treated well starting from the 2^nd^ well to the 10^th^ well. One hundred microliters of parasite mix without any drug was added to the 11^th^ well as a negative control, and the procedure was repeated for the rest of other extract/compounds. The plates were arranged in a modular chamber and gassed for 5 min with gas mixture of 5% oxygen, 5% carbon dioxide, and 90% nitrogen and then kept at 37°C for 72 h.

#### 2.3.4. SYBR Green Assay

The plates were harvested after 72 h, and the assays were paused by adding 100 *μ*l lysing buffer containing SYBR green to each well and was thoroughly and gently mixed to avoid production of bubbles. The plates were then incubated in the dark for 30-60 minutes before reading the assay using FLUOstar OPTIMA Fluorometer plate reader with control software version 2.20 at 470 nm and 520 nm wavelengths.

#### 2.3.5. Cytotoxicity Assay

One hundred microliters of each diluted compounds with concentrations ranging from 6.25 *μ*g/ml to 100 *μ*g/ml was put in triplicate wells of a 96-well microtiter plate. Following that, 100 *μ*l of uninfected red blood cells was put to each well. Compound, culture medium, and uninfected red blood cells were subtracted from the optical densities by running control experiments for each parameter independently alongside the main experiment. The plates were then incubated for 72 hours at 37°C in a humidified incubator with 5% O_2_ and CO_2_ before 20 *μ*l of 2.5 mg/ml MTT (in phosphate-buffered saline) solution was added to each well, and the plate was incubated for another 2 hours. After incubation, aliquots of culture medium (150 *μ*l) were taken and discarded from each well, and 200 *μ*l of Triton X-100 in acidified isopropanol was added to each well to dissolve any formazan produced. The plates were then maintained at room temperature in the dark for 24 hours before the optical densities of the wells were measured using a plate reader at 570 nm. The concentrations at which 50% cytotoxicity occurred (CC_50_ values) were obtained by using Microsoft Excel Version 2010. The CC_50_ values were compared to the standard values [16].

### 2.4. Statistical Analysis

Each compound was tested in triplicate concentration that inhibits asexual *Plasmodium falciparum* parasite by 50% (IC_50_) estimated from dose-response curves by nonlinear regression analysis using GraphPad Prism version 7.0 software (GraphPad software, San Diego, CA, USA). Cytotoxicity analysis was carried out using Microsoft Excel Version 2010.

## 3. Discussion of Results

### 3.1. Chemistry

In this study, a number of phenylhydrazones with N-substituted groups were resynthesized. The preferred technique for producing 2,4-dinitrophenylhydrzones (Brady's product) is Brady's condensation reaction between carbonyls and 2,4-dinitrophenylhydrazine [17]. The structures of the synthesized phenylhydrazones were validated by the spectroscopic data. Notably, the deshielding action of the nearby tertiary nitrogen caused the imine proton to give off a singlet at 8.86. Additionally, the tautomerism that can occur between the imine nitrogen and the tertiary carbon may be the cause of the additional quaternary carbon. Figure S1 to Figure S12 show the compounds' ^1^H and DEPTQ NMR spectra.

### 3.2. Molecular Docking Studies

**Figure 4:**
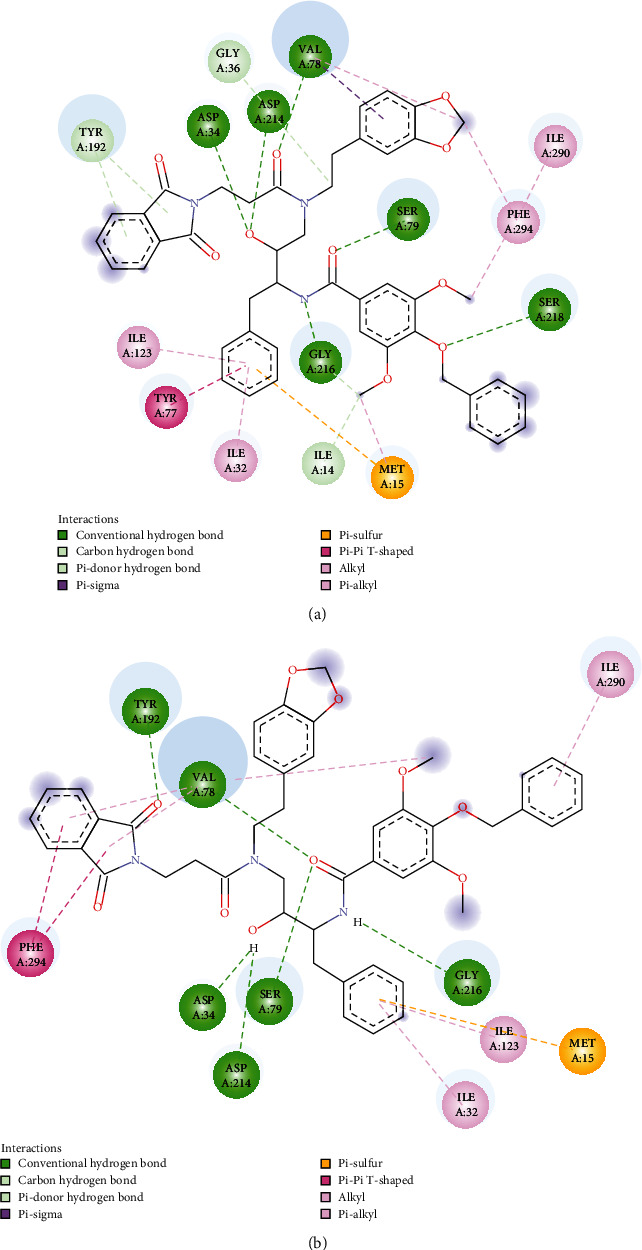
(a) Interactions of 1LF3 with EH58 from http://rcsb.org/pdb and (b) 1LF3 with EH58 after redocking, showing hydrogen bonds of distances less than 3.5 Å.

Molecular docking is an *in silico* drug design means of identifying optimized poses and predicting affinity of ligands in a protein [18–20]. This simulation was performed to explore the binding poses of the ligands in the binding pocket of the key enzymes from *P. falciparum* used in the study. The grid coordinates and box dimensions of 1LF3, 1LS5, and 6SSZ used for blind docking and local docking are presented in Figure 4 and Table 1. Redocking of EH58 with ILF3 was performed to validate the docking protocol (Figure 4). Root mean square deviation (RMSD) between 1.1 and 1.8 Å for the redocking protocol was obtained in PyMOL (Figure S13). The details of hydrophobic and hydrogen bond interactions of EH58 and 1LF3 are shown in Figure 4. The ligand binding potentials of the phenylhydrazones can also be seen in Table 2. Thus, the phenylhydrazones had high affinities for the regulatory proteins of *Plasmodium falciparum* when compared to chloroquine (control drug).

**Figure 5:**
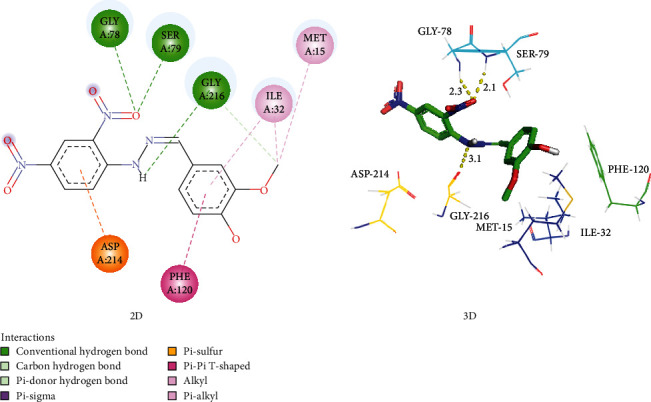
Interaction of plasmepsin IV with PHN3, with hydrogen bonds shown as green-dotted lines (2D) and yellow-dotted lines (3D).

**Figure 6:**
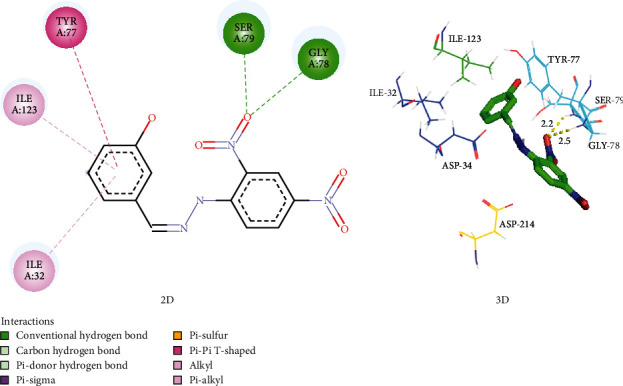
Interaction of plasmepsin IV with PHN6, with hydrogen bonds shown as green-dotted lines (2D) and yellow-dotted lines (3D).

The binding pocket of the plasmepsin IV protein contains the catalytic Asp34, Ile75, Tyr77, Gly78, Ser79, Ile114, Leu131, 1le133, Asp214, Thr217, and Leu292 residues [21]. An examination of the docked outputs of PHN3 and PHN6 with this protein suggests that they form at least two hydrogen bonds of distances less than 3.5 Å with these residues as depicted in Figures 5 and 6. Pepstatin A, a potent inhibitor of plasmepsin proteins, has similar hydrogen bond interaction with the enzyme. These interactions were concentrated on the nitro groups on the benzene rings of PHN3 and PHN6 as well as the hydroxyl groups on the implicated residues.

The flap residues Val78 and Ser79 present in the active site of Plm II form hydrogen bonds with PHN4 via the main-chain nitrogen atom of Val78 at 2.3 Å and the side-chain hydroxyl group of Ser79 at 2.1 Å bond length. In addition, the hydroxyl group of Tyr192 residue of Plm II forms hydrogen bonds with the PHN4 at 2.5 Å. The inhibitor EH58 and the plasmepsin II protein interact in ways that are consistent with these relationships (Figure S14 and Table S1). The phenylhydrazones demonstrated higher affinities for plasmepsin and falcipain-2 protease in comparison to chloroquine; hence, binding of these compounds to these proteins could be responsible for the *in vitro* activity (Figures 5 and 6 and Table 2).

**Figure 7:**
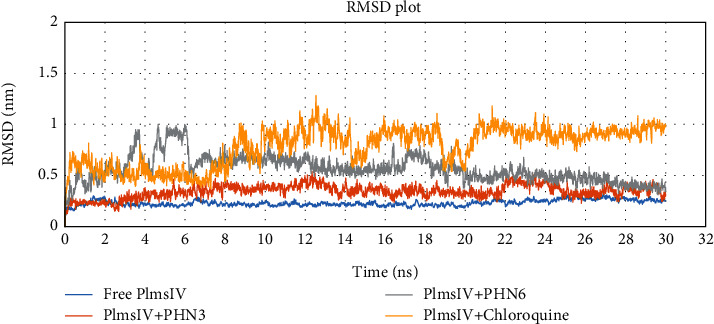
Analysis of root mean square deviation of free protein and bounded protein; free protein (Plm IV) in blue color, control drug (chloroquine) and protein in yellow color, and selected ligands (PHN3 and PHN6) and protein in orange and grey colors, respectively.

We further carried out molecular dynamic (MD) simulation to validate the conformational stability of the studied phenylhydrazones upon binding with Plm IV protein. The first round of MD simulation was conducted for Plm IV Apo enzyme to check its stability throughout the entire 30 ns simulation. The best protein-ligand complexes obtained from molecular docking studies were selected for the second round of the 30 ns MD simulation. This simulation was carried out to assess the stability of free Plm IV upon binding of these ligands. The compounds PHN3 and PHN6 recorded the best IC50 values from the *in vitro* studies (Table 3 and Figure S17 and S18) and displayed optimal interactions with crucial amino acid residues of plm IV protein (Figures 5 and 6) from molecular docking studies. Hence, the best poses of PHN3, PHN6, and chloroquine (control drug) were chosen for the 30 ns MD simulation. The root mean square deviation (RMSD) for the backbone atoms of Plm IV free form and ligand-bound complexes was calculated over a 30 ns simulation, and the results are presented in Figure 7. The RMSD of Plm IV-PHN3 and Plm IV-PHN6 remained below 2 nm throughout the simulation; hence, the protein-ligand complexes were stable and did not induce structural instability to the proteins.

**Figure 8:**
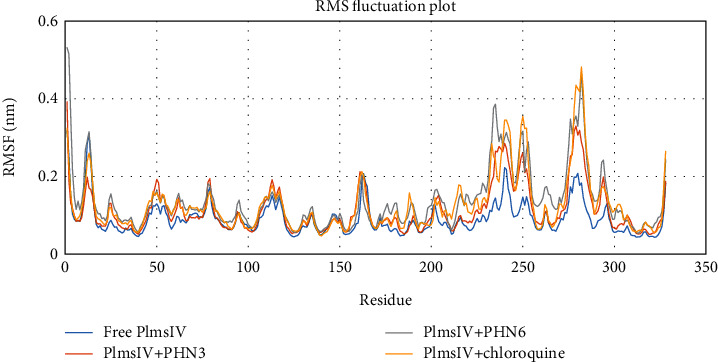
Analysis of RMSF of free protein and bounded protein; free protein (Plm IV) in blue color, control drug (chloroquine) and protein in yellow color, and selected ligands (PHN3 and PHN6) and protein in orange and grey colors, respectively.

**Figure 9:**
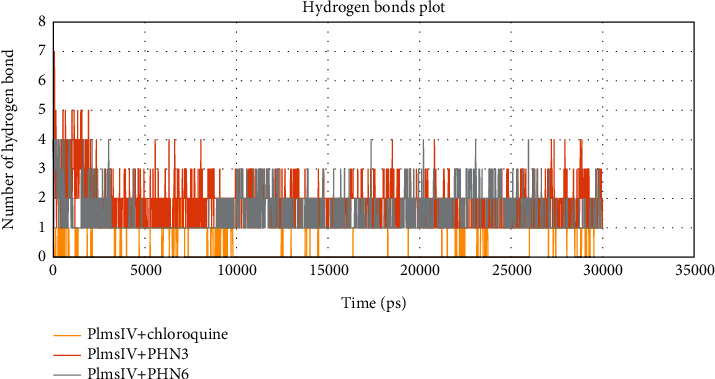
Analysis of hydrogen bonds of bounded protein; control drug (chloroquine) and protein in yellow color and selected ligands (PHN3 and PHN6) and protein in orange and grey colors, respectively.

We also explored the flexibility of each amino acid residue upon binding of PHN3, PHN6, and chloroquine to Plm IV protein by calculating and plotting the root mean square fluctuation (RMSF) of amino acid residues, and this is illustrated in Figure 8. We observed that the residues at the binding site of Plm IV-PHN3 and Plm IV-PHN6 complexes did not show any major fluctuation, and the ligands (PHN3 and PHN6) remained in close contact with the active sites of Plm IV. This was evident from their small RMS fluctuation under 1 nm throughout the entire period of the MD simulation. The number of hydrogen bonds formed between Plm IV and the ligands was calculated with the help of the gmx hbond inbuilt module. A maximum number of seven hydrogen bonds and four hydrogen bonds were observed with Plm IV-PHN3 and Plm IV-PHN6, respectively. The gmx hbond analysis of Plm IV-chloroquine indicated that it formed the least number of hydrogen bonds with Plm IV throughout the simulation period as depicted in Figure 9. At least one hydrogen bond was observed for Plm IV-PHN3 and Plm IV-PHN6 throughout the simulation; hence, PHN3 and PHN6 formed more stable hydrogen bonds with the binding sites of Plm IV when compared to chloroquine.

**Figure 10:**
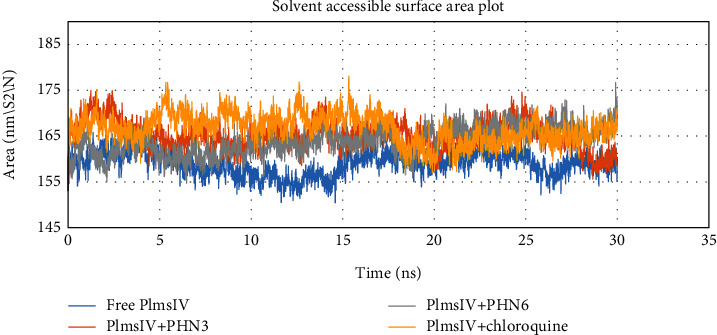
Analysis of total solvent accessible surface area of free protein and bounded protein; free protein (Plm IV) in blue color, control drug (chloroquine) and protein in yellow color, and selected ligands (PHN3 and PHN6) and protein in orange and grey colors, respectively.

**Figure 11:**
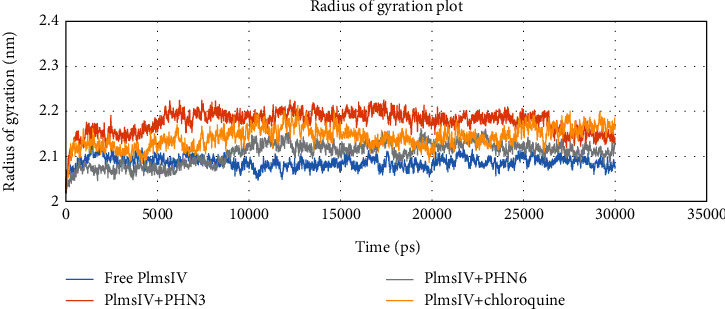
Analysis of radius of gyration of free protein and bounded protein; free protein (Plm IV) in blue color, control drug (chloroquine) and protein in yellow color, and selected ligands (PHN3 and PHN6) and protein in orange and grey colors, respectively.

The ligand-bound complexes also showed less deviation from the free Plm IV protein from total solvent accessible surface area analysis and were found to be in the region of 155 and 175 nm^2^ (Figure 10). Analysis of radius of gyration plot in Figure 11 showed that PHN3 and PHN6 caused less fluctuation when bound to the Plm IV relative to chloroquine; hence, the binding of the PHN3 and PHN6 to Plm IV does not have an impact on its compactness. Our selected ligands (PHN3 and PHN6) performed better when compared to chloroquine (control drug) regarding binding affinity from molecular docking studies and molecular stability from molecular dynamic simulations with the regulatory proteins of *Plasmodium falciparum*.

### 3.3. ADME Prediction and Toxicity

A portion of unsuccessful clinical trials have been connected to the drug candidates' poor absorption, distribution, metabolism, and excretion properties [22]. *In silico* prediction of the physicochemical, pharmacokinetic, and toxicity profiles of the synthesized phenylhydrazones was performed. According to the SwissADME study in Table 4, four of the six synthesized phenylhydrazones (PHN1, PHN4, PHN5, and PHN6) demonstrated strong gastrointestinal absorption and good oral bioavailability by recording topological polar surface area (TPSA) values of 140.2 Å (Table 5) according to the SwissADME analysis. Thus, the four phenylhydrazones have high oral bioavailability due to their strong cell permeability and transport capabilities. Additionally, it was projected that these phenylhydrazones would have excellent percentage (%) absorption between 62 and 69%, hence are close to drug-likeness. Once more, each of them conforms to Lipinski's rule of five for orally active medicines and has the potential to be developed into a medication candidate because none of them revealed a violation of the rule. The studied phenylhydrazones do not cross the blood-brain barrier (BBB) and are not P-glycoprotein (P-gp) substrates, according to *in silico* pharmacokinetic analysis. The likelihood that P-glycoprotein will efflux those ligands out of the cell, resulting in poor bioavailability, is therefore quite low. PHN3 and PHN6 have high ligand lipophilicity indexes (LLE), which was consistent with their maximum potency. This indicates that the ligands have a 1,000 times greater affinity for their target than for 1-octanol, indicating that the potency of PHN2, PHN3, PHN5, and PHN6 is thought to be connected to their lipophilicity.

Toxicity profile of all the phenylhydrazones from Pro Tox II showed that they are not cytotoxic. However, they exhibited mutagenicity and hepatotoxicity potentials (Table S2). They were therefore labelled as category IV compounds according to globally harmonized system of classification of labelling chemical toxicity grading. Further optimization could therefore focus on improving the toxicity profile of these phenylhydrazones.

### 3.4. Biology

It has been suggested that a library of phenylhydrazone derivatives with different substituents on the aromatic ring is a prospective collection of drugs for the treatment of drug-resistant malaria [23, 24].

The phenylhydrazone scaffold was maintained throughout the design process, but the substituent groups were varied. The substituent that is immediately connected to the imine has significance on the potency of the compounds against *P. falciparum*, according to the docking studies. The potency increases with the number of electron-donating groups, such as hydroxy, methyl, or methoxy on the N-substituted aromatic group. In terms of suppressing parasitemia, compounds with no hydrogen linked to the imine nitrogen, such as PHN1, or with disubstituted hydroxy groups, such as PHN2, had low potency. Compounds made from aldehyde substrates with disubstituted diversification, on the other hand, showed a more potent deadly impact on the parasite. The aforementioned findings became even more apparent when PHN3 (IC_50_: 6.600.0334 (3D7)) and PHN6 (IC_50_: 1.2860.0521 (3D7)), as shown in (Table 3 and Figure S15 and Figure S16) both showed higher potency than their respective positional vector isomers.

The IC_50_ values did not actually match the compounds' lipophilicity. For example, the *para* methoxy group in PHN1 had a considerable rise in clogP, but PHN1 with the greatest cLogP had the lowest activity, demonstrating that lipophilicity may not play a significant role in predicting antimalarial activity of the compounds. The Lipinski's rule of five must be applied when evaluating these compounds as potential oral drug candidates. From Table 5, it is clear that all of the compounds PHN1-PHN6, which ranged in molecular weight from 286.24 to 420.37, complied with Lipinski's rule of five. When it comes to cytotoxicity, the ability of a potential clinical candidate to discriminate between the pathogen and the host is crucial and is often taken into account in the preclinical phases [25, 26]. PHN6 and PHN3 had some of the greatest selectivities with the least selective molecule as PHN1, which included two aromatic groups (Table 3). This could suggest that there is a link between lipophilicity and toxicity; hence, there is a need for further optimization to improve the safety profile.

## 4. Conclusion

We have resynthesized the existing library of phenylhydrazones designed to display the antimalarial potency but could not avoid associated predicted toxicity issues caused by the imine group. We have described the SAR and optimizations for potency and ADME properties. Exploratory SAR has suggested electron-donating groups on *meta* and *para* vector positions as suitable replacements for the monosubstituted rings. With respect to CQ-sensitive and particularly CQ-resistant strains of *P. falciparum*, these compounds have strong *in vitro* activity. The high activity of the most potent phenylhydrazones is linked with binding to the Arg307, Tyr272, and Asn13 (plasmepsin II) and Gly216, Gly78, and Ser79 (plasmepsin IV), respectively, for PHN3 and Arg307, Lys163, and Lys327 (plasmepsin II) and Ser78 and Ser79 (plasmepsin IV) for PHN6 in the hinge region of the alpha chain, making the haemoglobin susceptible for further degradation. With the phenylhydrazones not able to cross the BBB and poor P-glycoprotein (P-gp) substrates coupled with a good absorption of 62% to 69% and good selective toxicity, this study has provided information for hit expansion and H2L optimization for better ADME-Tox profile.

## Figures and Tables

**Table 1:** Grid coordinates and box dimensions of 1LF3, 1LS5, and 6SSZ used for blind docking and local docking.

Protein	PDB ID	Grid coordinates and box dimensions											
		Blind docking						Local docking					
		Center (*x*, *y*, *z*) (Å)			Box size (*x*, *y*, *z*) (Å)			Center (*x*, *y*, *z*) (Å)			Box size (*x*, *y*, *z*) (Å)		
Plm II	1LF3	16.00	7.00	25.00	60.00	60.00	60.00	16.22	6.85	27.61	20.50	23.64	24.74
Plm IV	1LS5	-26.0	38.00	41.00	60.00	60.00	60.00	-26.6	38.15	41.29	20.50	23.64	24.74
PfFP2	6SSZ	25.00	-35.0	15.00	60.00	60.00	60.00	16.72	-40.4	5.05	20.30	16.30	15.61

**Table 2:** Results of blind and local docking of PHN1, PHN2, PHN3, PHN4, PHN5, and PHN6 with plasmepsin II (PDB ID: 1LF3), plasmepsin IV (PDB ID: 1LS5), and falcipain-2 protease (PDB ID: 6SSZ).

Compounds	Binding energy (kcal/mol)											
	Plasmepsin II (1LF3)				Plasmepsin IV (1LS5)				Falcipain-2 (6SSZ)			
	B1	B2	L1	L2	B1	B2	L1	L2	B1	B2	L1	L2
PHN1	-8.3	-7.9	-8.0	-8.0	-8.4	-8.3	-8.3	-8.3	-7.3	-6.9	-7.4	-7.2
PHN2	-7.5	-7.3	-7.6	-7.7	-7.1	-7.1	-7.5	-7.4	-6.8	-6.6	-6.9	-6.9
PHN3	-7.3	-7.3	-7.2	-7.3	-7.4	-7.2	-7.7	-7.7	-7.1	-7.1	-6.8	-6.9
PHN4	-7.5	-7.6	-7.6	-7.6	-7.8	-7.6	-7.4	-7.6	-6.8	-6.3	-6.7	-6.6
PHN5	-7.4	-7.3	7.4	-7.4	-7.6	-7.6	-7.8	-7.6	-6.1	-6.0	-7.0	-7.0
PHN6	-7.3	-7.3	-7.6	-7.6	-7.6	-7.6	-7.8	-7.8	-6.5	-6.2	-7.1	-7.1
Chloroquine	-6.3	-6.2	-6.2	-6.2	-6.4	-6.5	-6.5	-6.4	-5.8	-5.9	-5.9	-5.8

B1 = first round of blind docking; B2 = second round of blind docking; L1 = first round of local docking; L2 = second round of local docking.

**Table 3:** Potency and selective toxicity values of the compounds.

No.	3D7 IC_50_ (*μ*M)	DD2 IC_50_ (*μ*M)	CC_50_ (*μ*M)	SI = CC_50_/IC_50_ for 3D7	SI = CC_50_/IC_50_ for DD2
PHN1	31.01 ± 0.37	34.89 ± 0.17	106.4	3	3
PHN2	14.12 ± 0.04	16.24 ± 0.17	119.2	8	7
PHN3	6.60 ± 0.03	2.415 ± 0.01	131.8	20	55
PHN4	18.41 ± 0.03	19.82 ± 0.07	131.1	7	7
PHN5	19.02 ± 0.02	6.591 ± 0.06	111.5	6	6
PHN6	1.286 ± 0.05	5.121 ± 0.07	101.3	79	19
Artesunate	0.004688 ± 0.000017	0.005063 ± 0.000023	84.7	>1000	>1000
Chloroquine	0.0071 ± 0.542	0.0082 ± 0.522	>100	>1000	>1000

IC_50_: 50% inhibitory concentration; CC_50_: 50% cytotoxic concentration; CC_50_/IC_50_ = selectivity index (SI).

**Table 4:** Pharmacokinetic parameters of the compounds from SwissADME database.

Molecule	clogP	pIC_50_ 3D7	pIC_50_ Dd2	LLE 3D7	LLE Dd2	GI absorption	BBB permeant	Pgp substrate	In silico % ABS
PHN1	3.27	3.10	4.05	0.17	0.78	High	No	No	69.00
PHN2	0.57	3.57	4.39	3.00	3.82	Low	No	No	55.01
PHN3	1.29	4.74	5.18	3.45	3.89	Low	No	No	58.80
PHN4	1.39	4.24	4.20	2.85	0.04	High	No	No	69.00
PHN5	0.99	4.25	4.71	3.26	3.72	High	No	No	62.00
PHN6	0.99	5.37	4.77	4.38	3.78	High	No	No	62.00

Key: cLogP: calculated lipophilicity; pIC_50_: logarithm of 50% inhibitory concentration; LLE: ligand lipophilicity index; BBB: blood-brain barrier. The descriptors include MW: molecular weight; A: molar refractivity; HBD: hydrogen bond donor; HBA: hydrogen bond acceptor; RB: rotatable bonds; TPSA: topological polar surface area; L-Ro5: Lipinski's rule of five; L-Ro5: HBD ≤ 5, HBA ≤ 10, MW ≤ 500, log*P* ≤ 5.

**Table 5:** Physicochemical properties of the compounds from SwissADME database.

Molecule	MW	RB	HBA	HBD	A (cm^3^)	TPSA (Å^2^)	L-Ro5
PHN1	362.34	6	5	1	106.05	116.03	✓
PHN2	318.24	5	7	3	85.61	156.49	✓
PHN3	332.27	6	7	2	90.08	145.49	✓
PHN4	302.24	5	6	2	83.59	136.26	✓
PHN5	286.24	5	5	1	81.56	116.03	✓
PHN6	302.24	5	6	2	83.59	136.26	✓

MW: molecular weight; A: molar refractivity; HBD: hydrogen bond donor; HBA: hydrogen bond acceptor; RB: rotatable bonds; TPSA: topological polar surface area; L-Ro5: Lipinski's rule of five; L-Ro5: HBD ≤ 5, HBA ≤ 10, MW ≤ 500, log*P* ≤ 5.

## Data Availability

Data for this study is available in the archives of the Department of Pharmaceutical Chemistry, University of Health and Allied Sciences, Ho.

## Supplementary Materials

Supplementary Materials Figure S1: ^1^H NMR spectrum of compound PHN1. Figure S2: DEPT-Q NMR spectrum of compound PHN1. Figure S3: ^1^H NMR spectrum of compound PHN2. Figure S4: DEPT-Q NMR spectrum of compound PHN2. Figure S5: ^1^H NMR spectrum of compound PHN3. Figure S6: DEPT-Q NMR spectrum of compound PHN3. Figure S7: ^1^H NMR spectrum of compound PHN4. Figure S8: DEPT-Q NMR spectrum of compound PHN4. Figure S9: ^1^H NMR spectrum of compound PHN5. Figure S10: DEPT Q NMR spectrum of compound PHN5. Figure S11: ^1^H NMR spectrum of compound PHN6. Figure S12: DEPT-Q NMR spectrum of compound PHN6. Figure S13: validation of docking protocol: ligand superimposition: cocrystal in orange color and docked conformation in magenta color. Figure S14: interaction of plasmepsin II with PHN4, with hydrogen bonds shown as green-dotted lines in 2D and yellow-dotted lines in 3D. Figure S15: representation of IC_50_ values for 3D7. Figure S16: representation of IC_50_ values for Dd2. Table S1: hydrogen bond interaction of PHN1, PHN2, PHN3, PHN4, PHN5, and PHN6 with the molecular targets plasmepsin II (PDB ID: 1LF3), plasmepsin IV (PDB ID: 1LS5), and falcipain-2 protease (PDB ID: 6SSZ). Table S2: predicted toxicity profile of the compounds from Pro Tox II database.
