# Supplementary material for: N-Substituted Phenylhydrazones Kill the Ring Stage of Plasmodium falciparum
Source: Biomed Res Int. 2024 Feb 13;2024:6697728. doi: 10.1155/2024/6697728 (PMC10878751; doi:10.1155/2024/6697728)
Supplement: Supplementary Materials — Figure S1: 1H NMR spectrum of compound PHN1. Figure S2: DEPT-Q NMR spectrum of compound PHN1. Figure S3: 1H NMR spectrum of compound PHN2. Figure S4: DEPT-Q NMR spectrum of compound PHN2. Figure S5: 1H NMR spectrum of compound PHN3. Figure S6: DEPT-Q NMR spectrum of compound PHN3. Figure S7: 1H NMR spectrum of compound PHN4. Figure S8: DEPT-Q NMR spectrum of compound PHN4. Figure S9: 1H NMR spectrum of compound PHN5. Figure S10: DEPT Q NMR spectrum of compound PHN5. Figure S11: 1H NMR spectrum of compound PHN6. Figure S12: DEPT-Q NMR spectrum of compound PHN6. Figure S13: validation of docking protocol: ligand superimposition: cocrystal in orange color and docked conformation in magenta color. Figure S14: interaction of plasmepsin II with PHN4, with hydrogen bonds shown as green-dotted lines in 2D and yellow-dotted lines in 3D. Figure S15: representation of IC50 values for 3D7. Figure S16: representation of IC50 values for Dd2. Table S1: hydrogen bond interaction of PHN1, PHN2, PHN3, PHN4, PHN5, and PHN6 with the molecular targets plasmepsin II (PDB ID: 1LF3), plasmepsin IV (PDB ID: 1LS5), and falcipain-2 protease (PDB ID: 6SSZ). Table S2: predicted toxicity profile of the compounds from Pro Tox II database. [file 6697728.f1.docx]

**N-Substituted Phenylhydrazones Kill the Ring-Stage of *Plasmodium falciparum.***

Cedric Dzidzor Kodjo Amengor^1^, Prince Danan Biniyam^1^ , Abena Brobbey^2^, Francis Klenam Kekessie^3^, , Felix Kwame Zoiku^4^, Sherif Hamidu^5^, Patrick Gyan^2^, Billy Mawunyo Abudey^1^.

^1^Department of Pharmaceutical Chemistry, School of Pharmacy, University of Health and Allied Sciences, Ho-Ghana.

^2^Department of Pharmaceutical Chemistry, Faculty of Pharmacy and Pharmaceutical Sciences, Kwame Nkrumah University of Science and Technology, Kumasi-Ghana.

^3^Department of Chemistry and Biochemistry, Hattiesburg Campus, 118 College Drive Hattiesburg, the University of Sothern Mississippi, USA.

^4^Department of Epidemiology, Noguchi Memorial Institute for Medical Research, College of Health Sciences, University of Ghana, P.O. Box LG 581, Legon-Ghana

^5^Department of Clinical Pathology, Noguchi Memorial Institute for Medical Research, College of Health Sciences, University of Ghana, P.O. Box LG 581, Legon-Ghana.

Correspondence should be addressed to **Dr. Cedric Dzidzor Kodjo Amengor. Contact: Email: camengor@uhas.edu.gh. Tel: +233 (0) 246-456-764.**


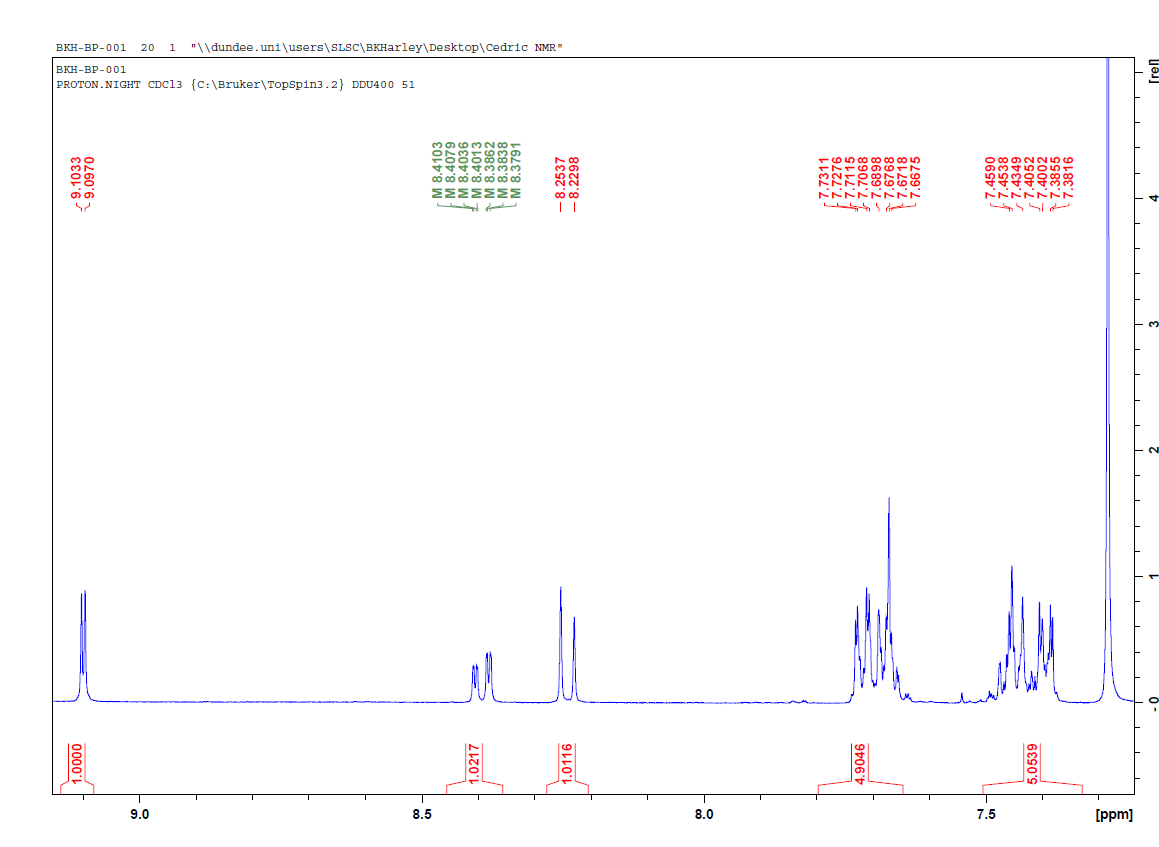


**S1 Fig. ^1^H NMR spectrum of compound PHN1**


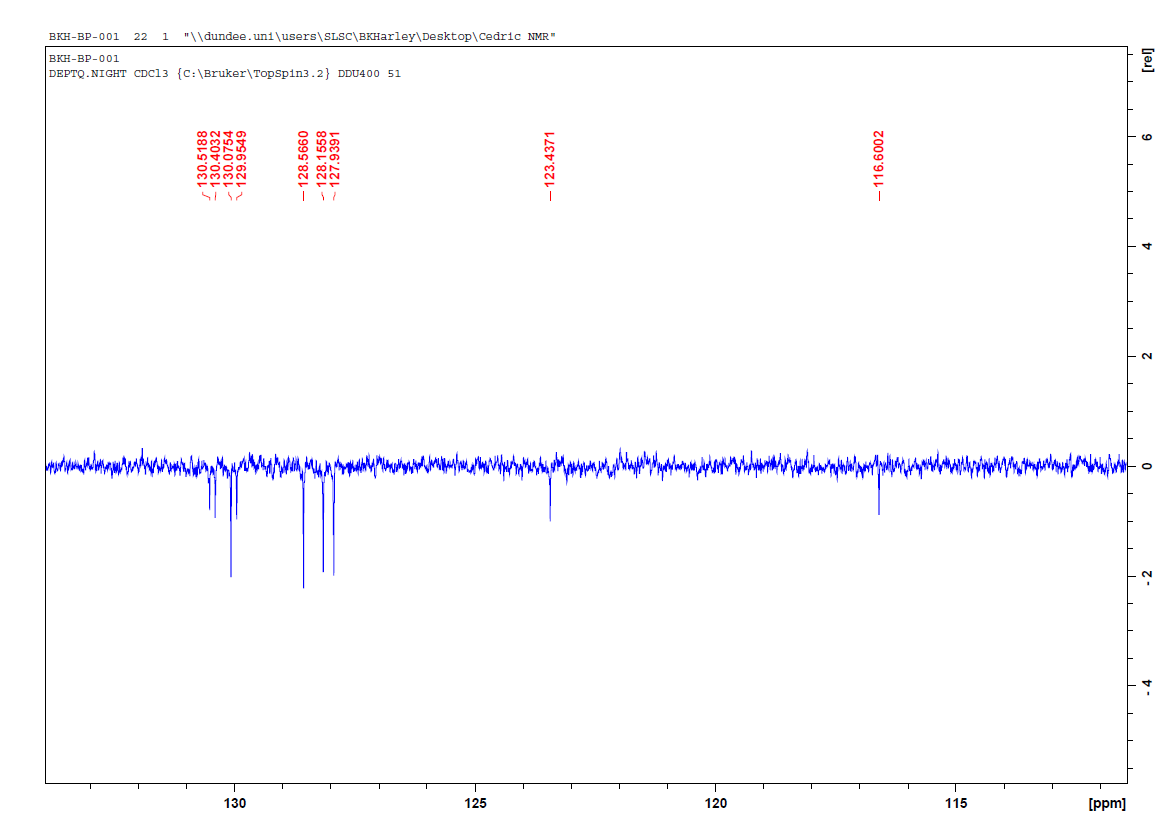


**S2 Fig. DEPT-Q NMR spectrum of compound PHN1**


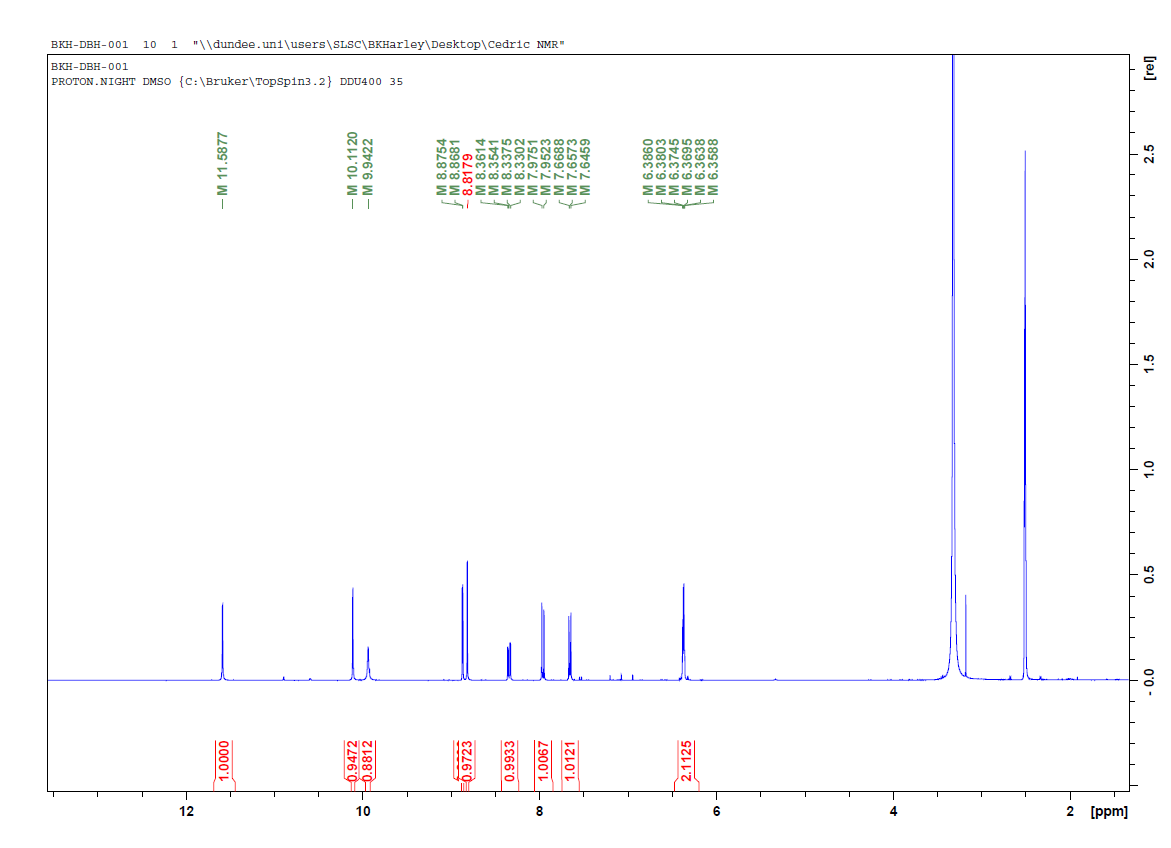


**S3 Fig. ^1^H NMR spectrum of compound PHN2**


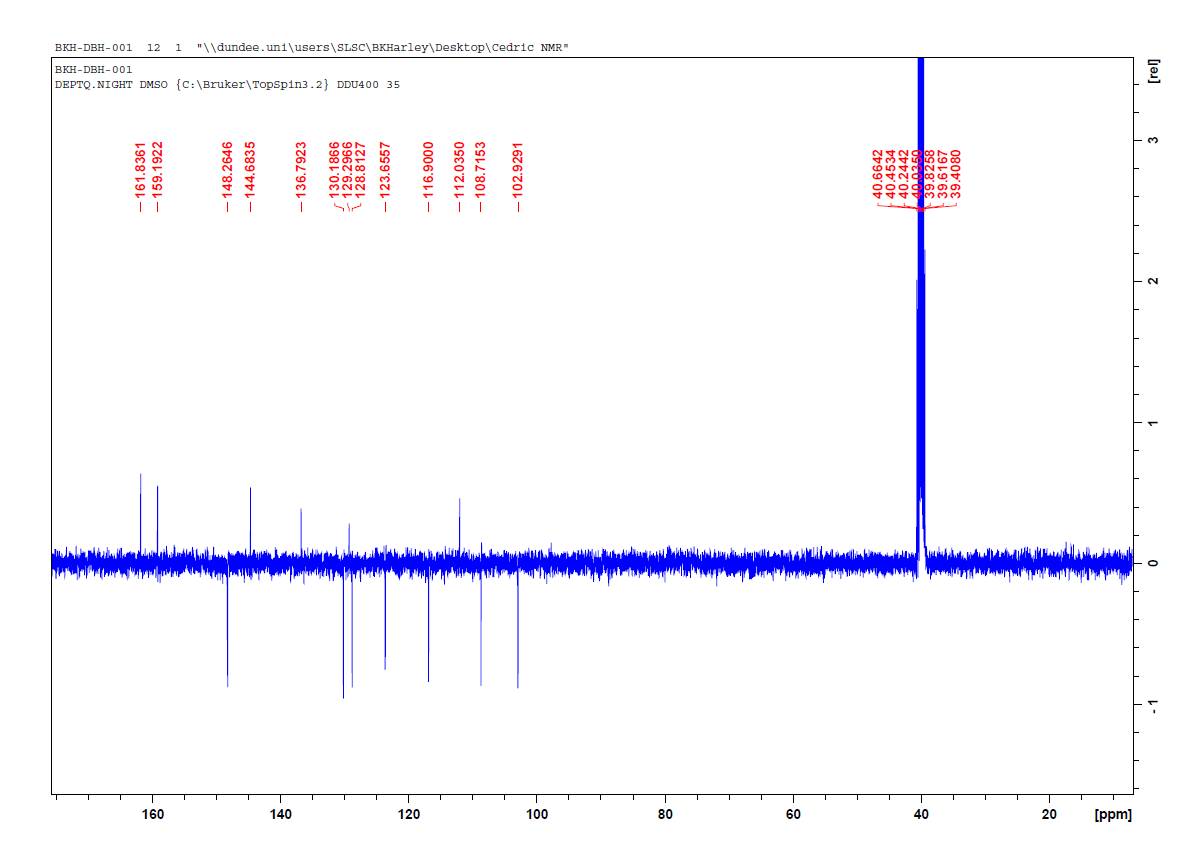


**S4 Fig: DEPT-Q NMR spectrum of compound PHN2**


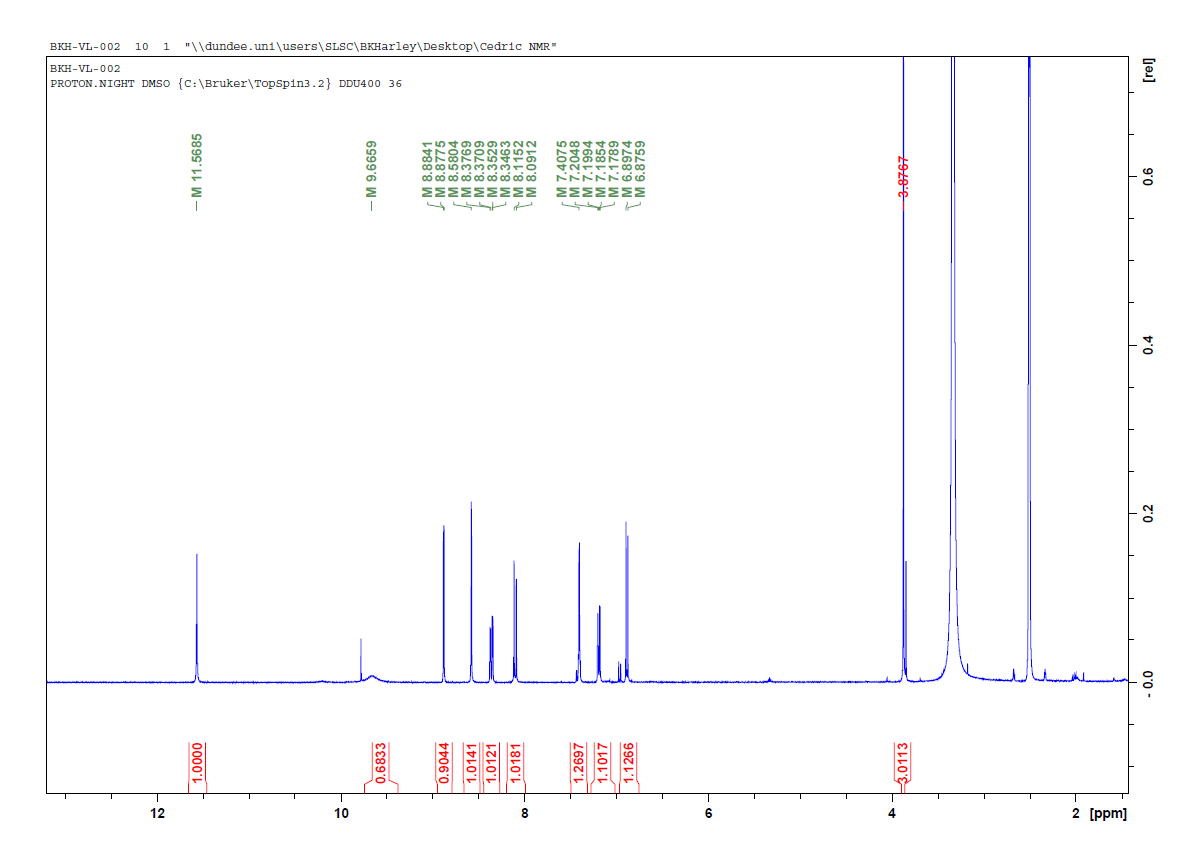


**S5 Fig. ^1^H NMR spectrum of compound PHN3**


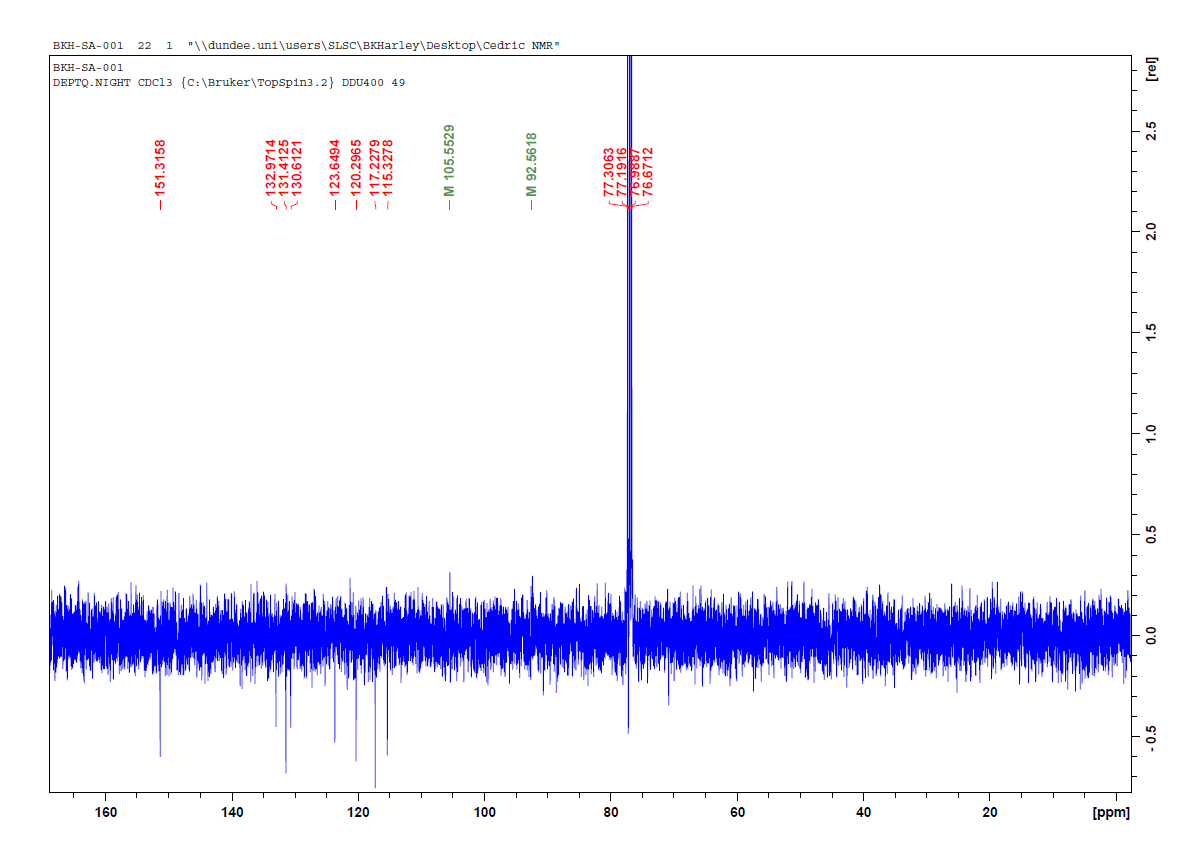


**S6 Fig. DEPT-Q NMR spectrum of compound PHN3**


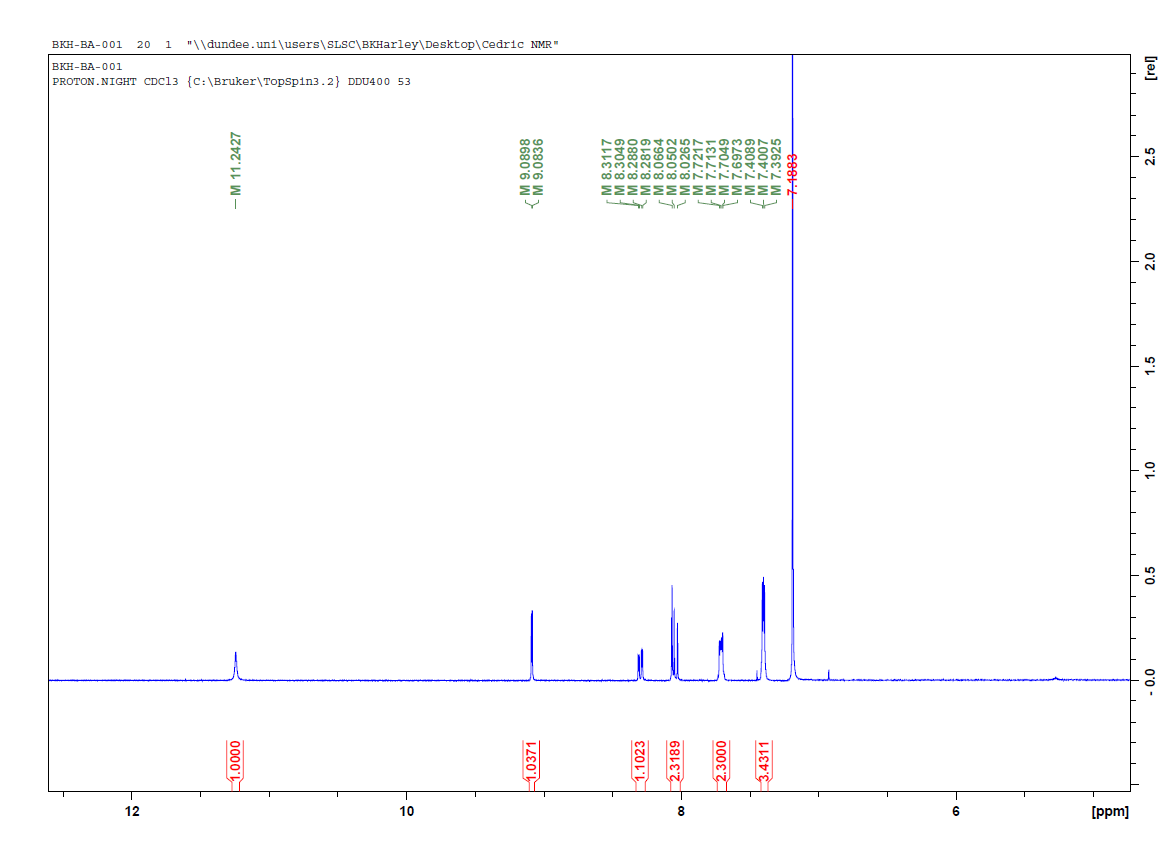


**S7 Fig. ^1^H NMR spectrum of compound PHN4**


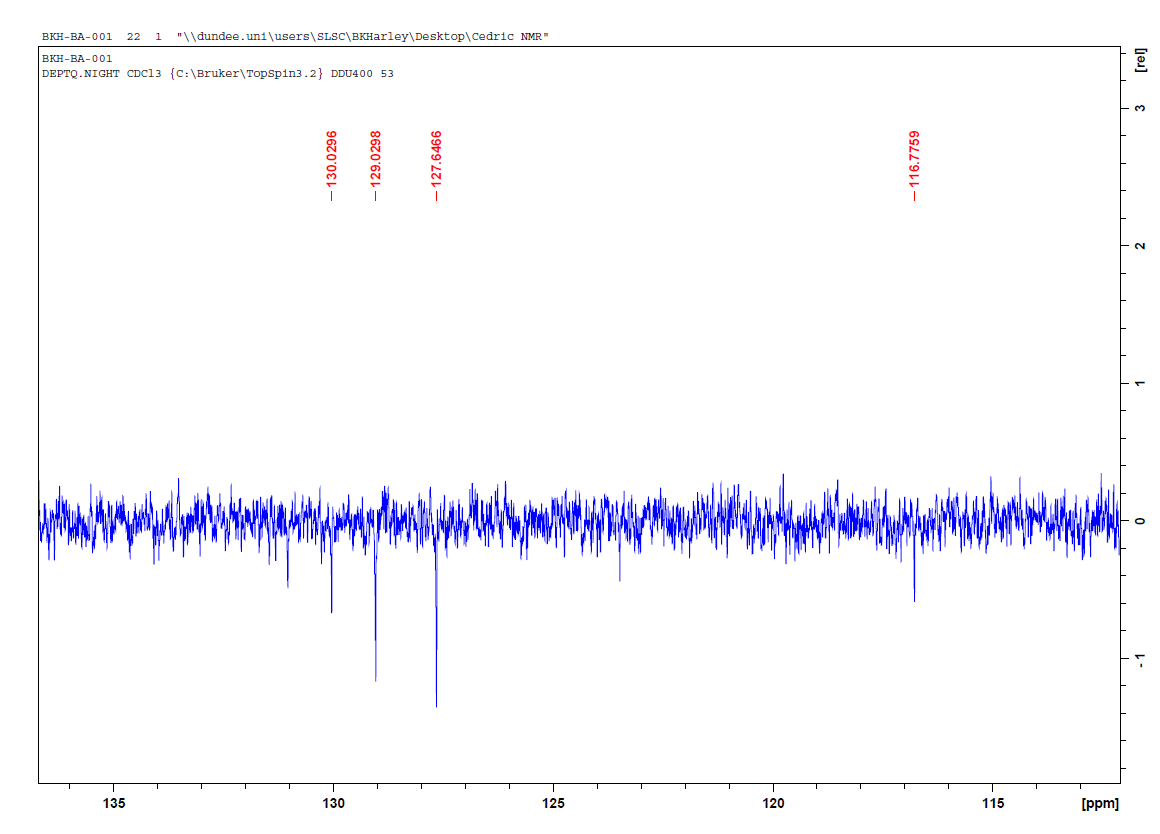


**S8 Fig. DEPT-Q NMR spectrum of compound PHN4**


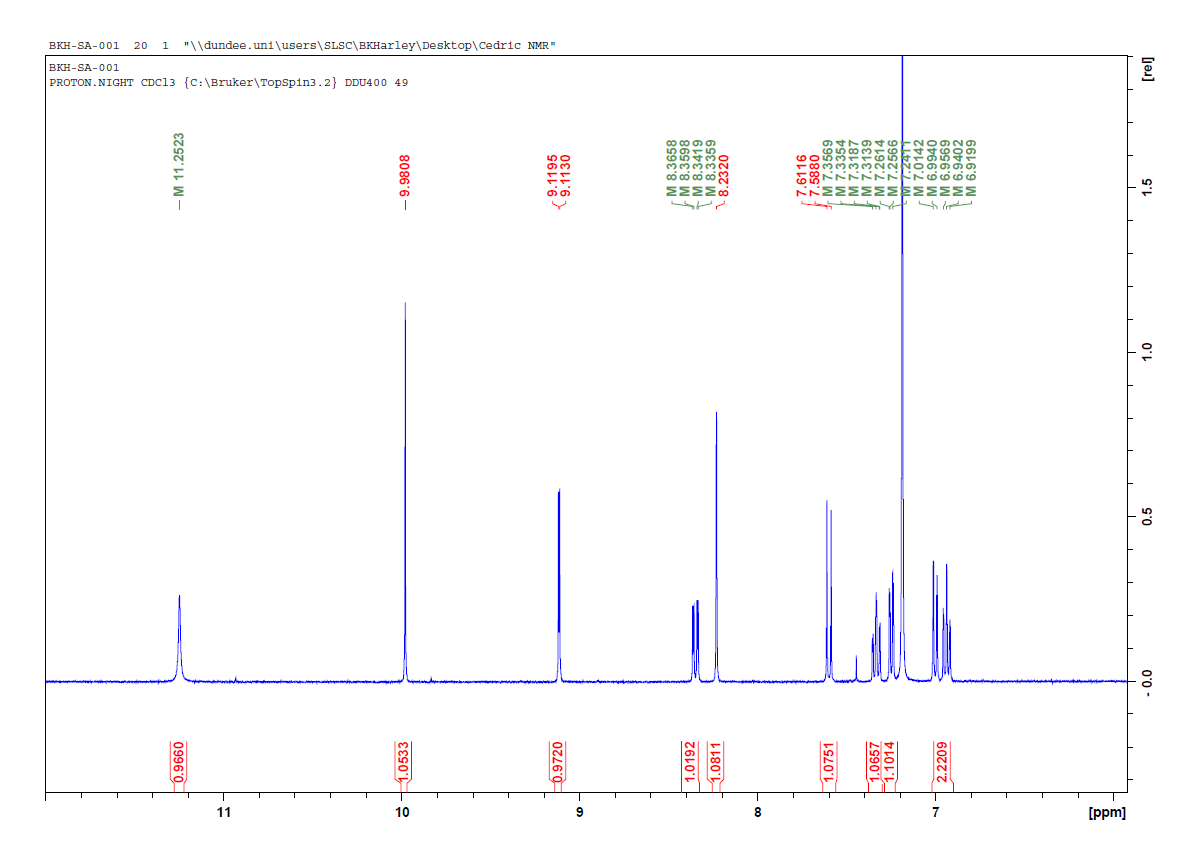


**S9 Fig. ^1^H NMR spectrum of compound PHN5**


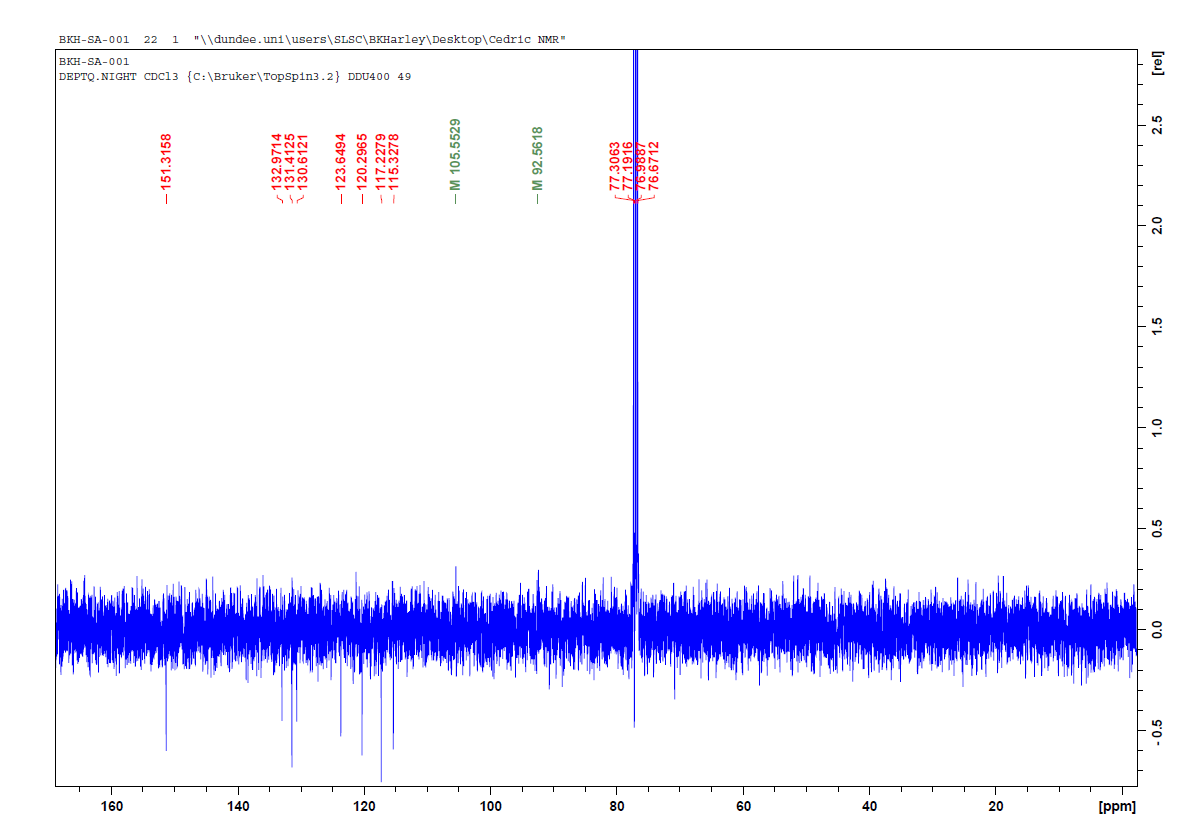


**S10 Fig. DEPT Q NMR spectrum of compound PHN5**


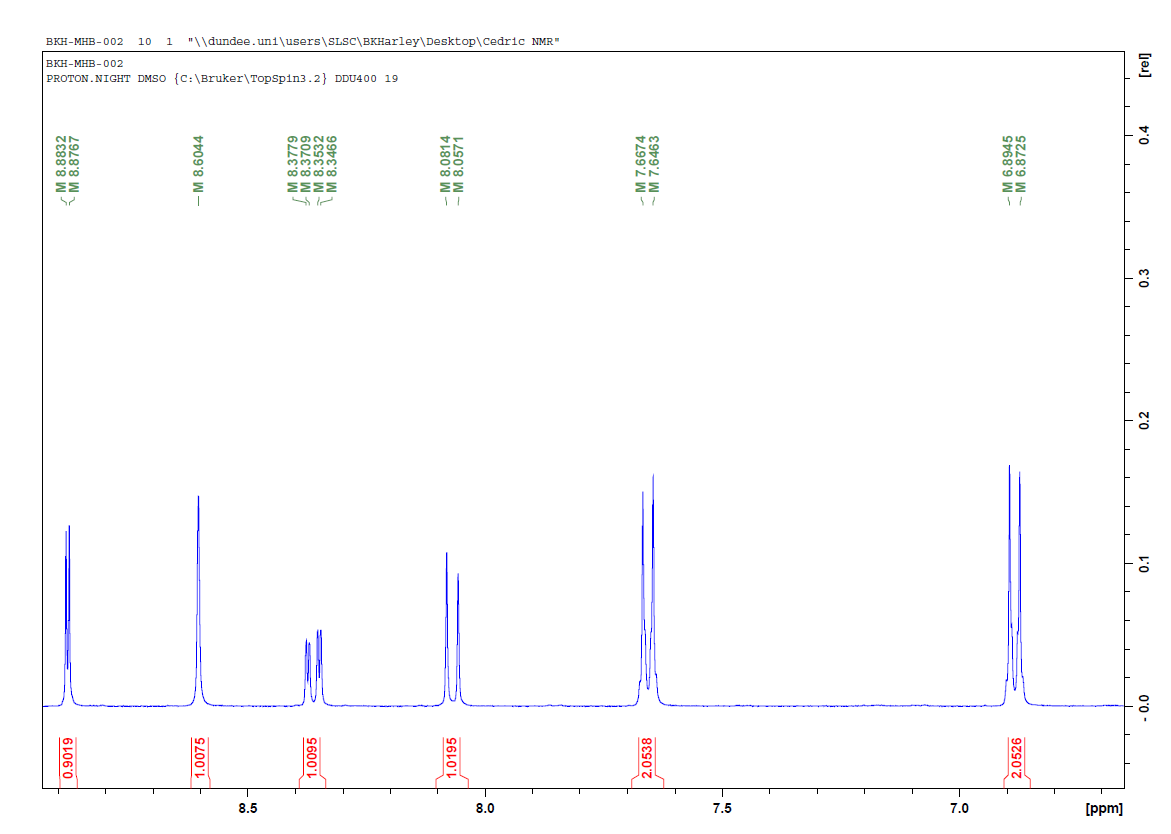


**S11 Fig. ^1^H NMR spectrum of compound PHN6**


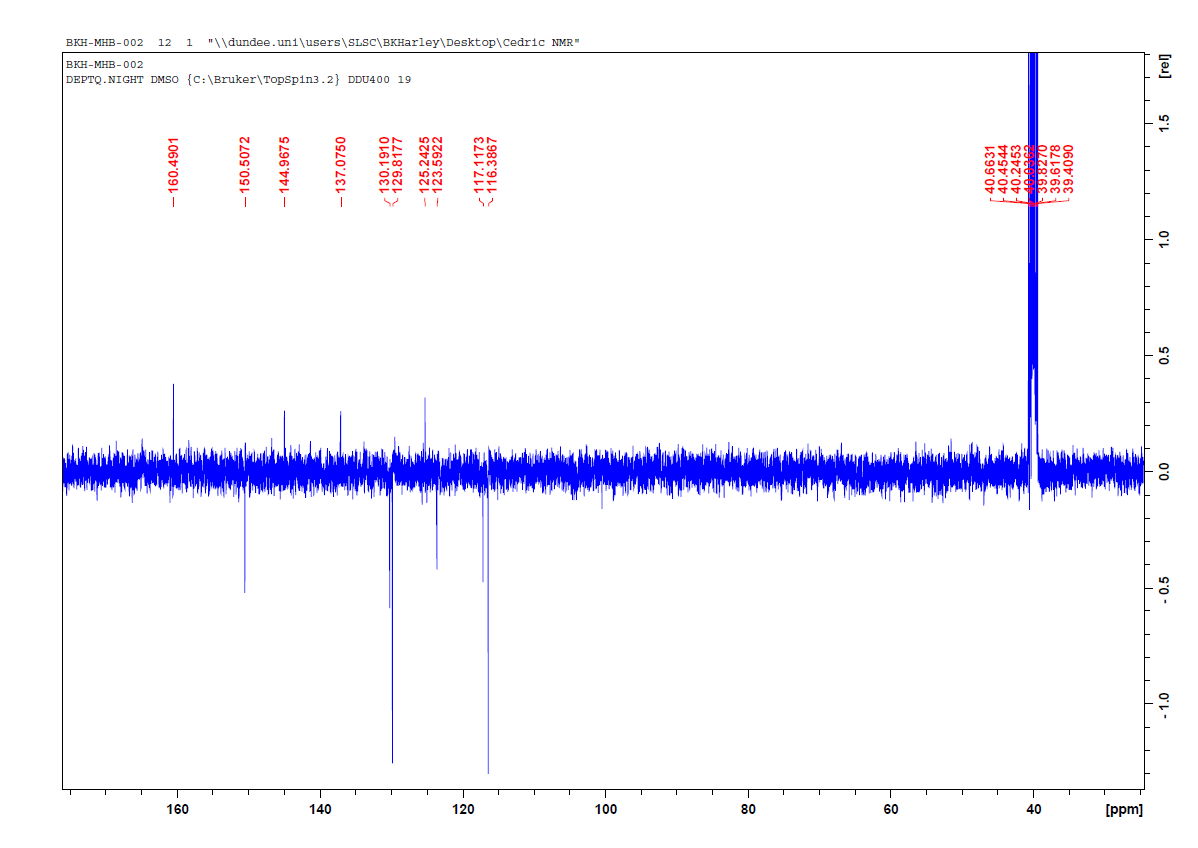


**S12 Fig. DEPT-Q NMR spectrum of compound PHN6**


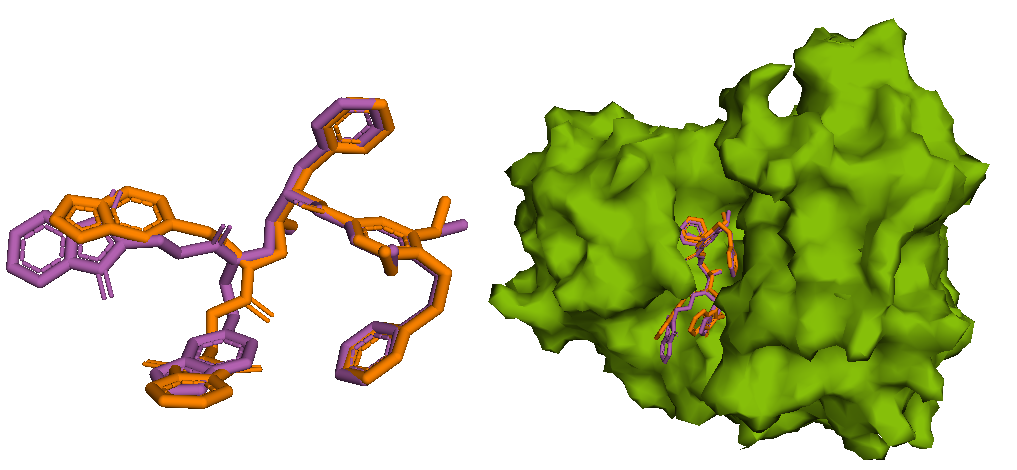


A B

S13 Fig. Validation of docking protocol: Ligand superimposition: co-crystal in orange colour and docked conformation in magenta colour


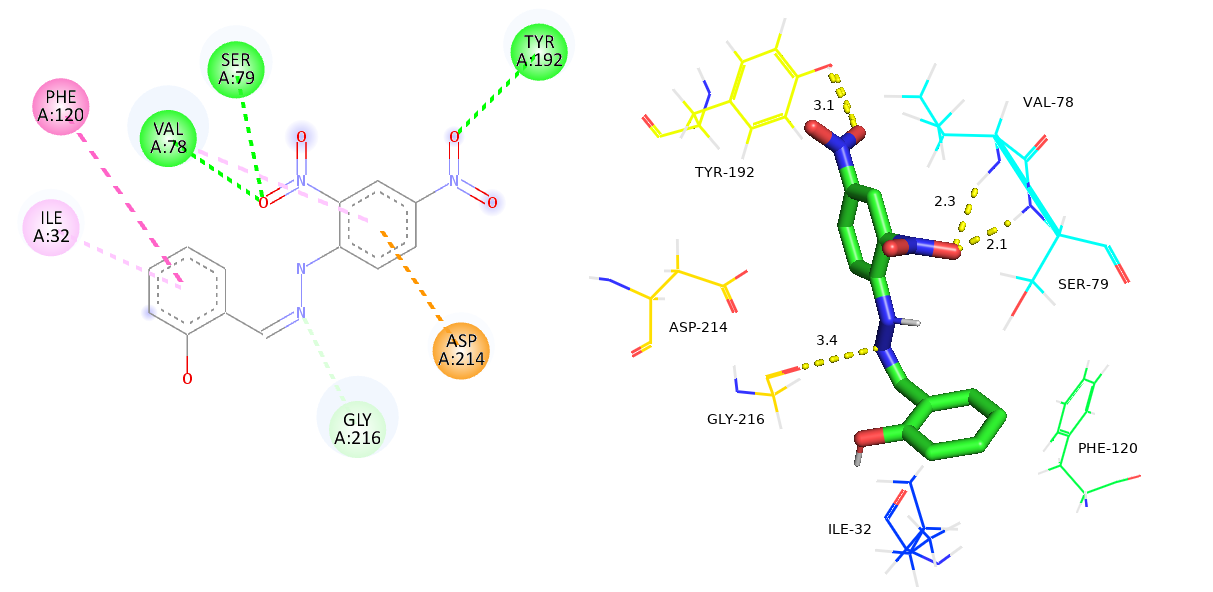


S14 Fig. Interaction of plasmepsin II with PHN4, with hydrogen bonds shown as green dotted lines in 2D and yellow dotted lines in 3D

S15 Fig. Representation of IC_50_ values for 3D7

S16 Fig. Representation of IC_50_ values for Dd2

S1 Table. Hydrogen bond interaction of PHN1, PHN2, PHN3, PHN4, PHN5 and PHN6 with the molecular targets plasmepsin II (PDB ID: 1LF3), plasmepsin IV (PDB ID: 1LS5) and falcipain 2 protease (PDB ID: 6SSZ)

| **Compound** | **Hydrogen bond interaction** | | |
| --- | --- | --- | --- |
|  | 1LF3 | 1LS5 | 6SSZ |
| PHN1 | Ser79, Ser218, Ala219 | Gly216, Ser79, Ser218, Thr217 | Gln19, Cys22 |
| PHN2 | Arg307, Asn13, His164, Lys326, Lys163 | Gly216, Gly78, Ser79, Ser218 | Asn156 |
| PHN3 | Arg307, Tyr272, Asn13 | Gly216, Ser79, Gly78 | Asn156 |
| PHN4 | Tyr192, Ser79, Val78, Gly216 | Ser218, Ser79 | Asn156 |
| PHN5 | Asn13, Arg307 | Ser79, Thr217 | Asn156 |
| PHN6 | Arg307, Lys163, Lys327 | Ser79, Gly78 | Asn156 |

S2 Table. Predicted toxicity profile of the compounds from Pro Tox II database

| Compound | Predicted Toxicity Class | Predicted LD50 (mg/kg) | Hepato toxicity | Mutagenicity | Cytotoxicity |
| --- | --- | --- | --- | --- | --- |
| PHN1 | 4 | 1000 | **Active** | **Active** | Inactive |
| PHN2 | 4 | 1000 | **Active** | **Active** | Inactive |
| PHN3 | 4 | 1000 | **Active** | **Active** | Inactive |
| PHN4 | 4 | 1000 | **Active** | **Active** | Inactive |
| PHN5 | 4 | 1000 | **Active** | **Active** | Inactive |
| PHN6 | 4 | 1000 | **Active** | **Active** | Inactive |
